# P-1826. Genomic Epidemiology and Clinical Characteristics of *Stenotrophomonas maltophilia* at an academic medical center

**DOI:** 10.1093/ofid/ofae631.1989

**Published:** 2025-01-29

**Authors:** Sahil Angelo, Lloyd Clarke, Ryan K Shields, Yohei Doi, Yohei Doi

**Affiliations:** University of Pittsburgh Medical Center, Pittsburgh, Pennsylvania; Antibiotic Management Program, UPMC Presbyterian Hospital, Pittsburgh, PA, Pittsburgh, Pennsylvania; University of Pittsburgh, Pittsburgh, Pennsylvania; Fujita Health University, Aichi, Aichi, Japan; Fujita Health University, Aichi, Aichi, Japan

## Abstract

**Background:**

*S. maltophilia* is an opportunistic pathogen in critically-ill and immunocompromised patients. Distinguishing between colonization and infection is challenging and genomic surveillance data are scarce. This study seeks to define the genomic epidemiology of *S. maltophilia* and associations between genomic groups (Gg) and clinical outcomes.

**Figure:**
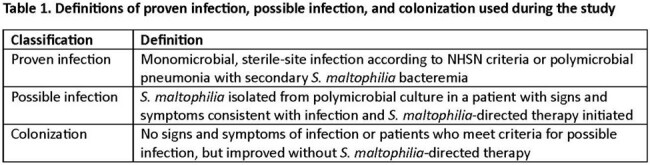

**Methods:**

Consecutive patients with *S. maltophilia* positive cultures were included from 10/21 – 12/23. Whole genome sequencing (WGS) was performed on the Illumina NextSeq 550 platform. Gg were identified by sequence type (ST). Cases were classified as proven infection, possible infection, or colonized (**Table 1**).

**Figure:**
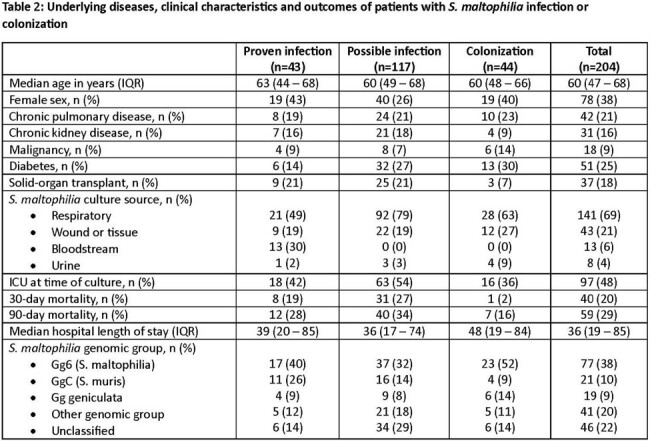

**Results:**

Across 204 patients, 21.1%, 57.3%, and 21.6% were classified as proven infection, possible infection, or colonized, respectively (**Table 2**). The median (IQR) age was 60 (47-68) years, 38% were women, and 18% were solid-organ transplant recipients. The overall 30- and 90-day mortality rates were 19.6% and 28.9%, respectively; 30-day mortality was higher for patients with proven or possible infection (24%) versus colonization (2%; *P*=0.004).

Significant diversity was identified across 470 *S. maltophilia* isolates sequenced (**Figure 1**). Using the first isolate from the 204 patients included in the study, 31 known STs were identified. ST5 and ST133 were most common (n=8 each). Isolates were classified into 9 different Cg (**Figure 2**); an additional 22% were unclassified. Gg6 (*S. maltophilia*) and GgC (*S. muris*) accounted for 38% and 10% of all isolates, respectively. Across syndromes, Gg6 was most common, accounting for 34% of proven/possible infections compared to 52% of colonization cases (*P*=0.03). GgC was more often associated with proven infection than colonization (*P*=0.05). Mortality rates were highest for Cg4 (43%) and lowest for Cg geniculate (5%).

**Figure 1.**
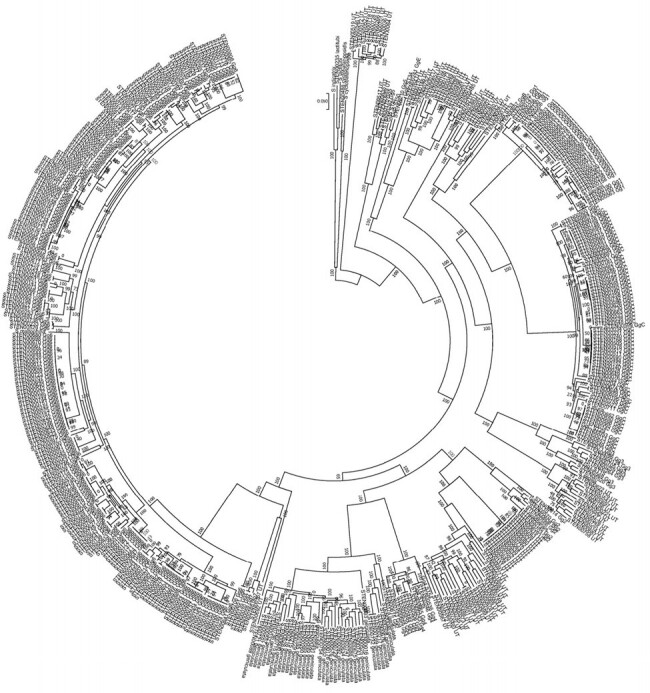
Phylogenetic tree of 470 S. maltophilia isolates collected at the study site

**Conclusion:**

*S. maltophilia* genomic groups are highly diverse and associated with varying clinical syndromes. Specific groups like GgC (*S. muris*) appear to be associated with high infection rates. Across Cg, mortality rates ranged from 5% to 43%, suggesting that differences may exist. These data support further molecular investigations into *S. maltophilia* Gg to help distinguish colonizing versus infecting isolates.

**Figure:**
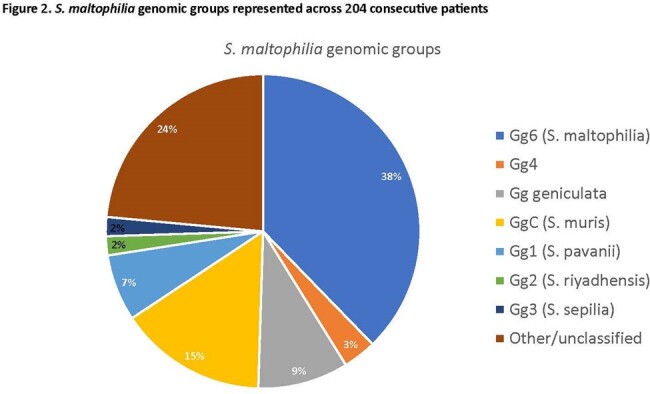

**Disclosures:**

**Ryan K. Shields, PharmD, MS**, Allergan: Advisor/Consultant|Cidara: Advisor/Consultant|Entasis: Advisor/Consultant|GSK: Advisor/Consultant|Melinta: Advisor/Consultant|Melinta: Grant/Research Support|Menarini: Advisor/Consultant|Merck: Advisor/Consultant|Merck: Grant/Research Support|Pfizer: Advisor/Consultant|Roche: Grant/Research Support|Shionogi: Advisor/Consultant|Shionogi: Grant/Research Support|Utility: Advisor/Consultant|Venatorx: Advisor/Consultant|Venatorx: Grant/Research Support **Yohei Doi, MD, PhD**, bioMerieux: Lecture fees|Entasis: Grant/Research Support|Fujifilm: Advisor/Consultant|Gilead Sciences: Advisor/Consultant|GSK: Advisor/Consultant|KANTO CHEMICAL CO.,INC.: Grant/Research Support|KANTO CHEMICAL CO.,INC.: Patent for genotyping kit|MeijiSeika Pharma: Advisor/Consultant|Moderna: Advisor/Consultant|MSD: Lecture fees|Pfizer: Advisor/Consultant|Shionogi & Co., Ltd.: Grant/Research Support|Shionogi & Co., Ltd.: Lecture fees

